# Identifying Causal Genes and Building Regulatory Networks in Crops Using the CisTrans-ECAS Method

**DOI:** 10.21769/BioProtoc.5578

**Published:** 2026-02-05

**Authors:** Yutong Yan, Luchang Ming, Weibo Xie

**Affiliations:** National Key Laboratory of Crop Genetic Improvement, Hubei Hongshan Laboratory, College of Informatics, Huazhong Agricultural University, Wuhan, China

**Keywords:** ****Keywords**:** Causal gene identification, Gene regulatory network, Transcriptome-wide association study (TWAS), Cis- and trans-expression components, Rice, Crop genetics

## Abstract

Pinpointing causal genes for complex traits from genome-wide association studies (GWAS) remains a central challenge in crop genetics, particularly in species with extensive linkage disequilibrium (LD) such as rice. Here, we present CisTrans-ECAS, a computational protocol that overcomes this limitation by integrating population genomics and transcriptomics. The method’s core principle is the decomposition of gene expression into two distinct components: a *cis*-expression component (*cis*-EC), regulated by local genetic variants, and a *trans*-expression component (*trans*-EC), influenced by distal genetic factors. By testing the association of both components with a phenotype, CisTrans-ECAS establishes a dual-evidence framework that substantially improves the reliability of causal inference. This protocol details the complete workflow, demonstrating its power not only to identify causal genes at loci with weak GWAS signals but also to systematically reconstruct gene regulatory networks. It provides a robust and powerful tool for advancing crop functional genomics and molecular breeding.

Key features

• Pinpointing causal genes with high precision: Integrates *cis-* and *trans*-expression components to distinguish true causal genes from LD artifacts, even for small-effect loci.

• Reconstructing gene regulatory networks: Uses gene expression as molecular traits to identify upstream regulators, revealing complex molecular regulatory pathways.

• Versatile and reproducible workflow: An R-based pipeline using PLINK and GCTA, applicable to rice and other species with population genomics and transcriptomics data.

• Experimentally validated reliability: The method successfully identified key genes *OsMADS17* and *SDT* that regulate rice spikelet number, with their regulatory relationship confirmed by molecular experiments.

## Graphical overview


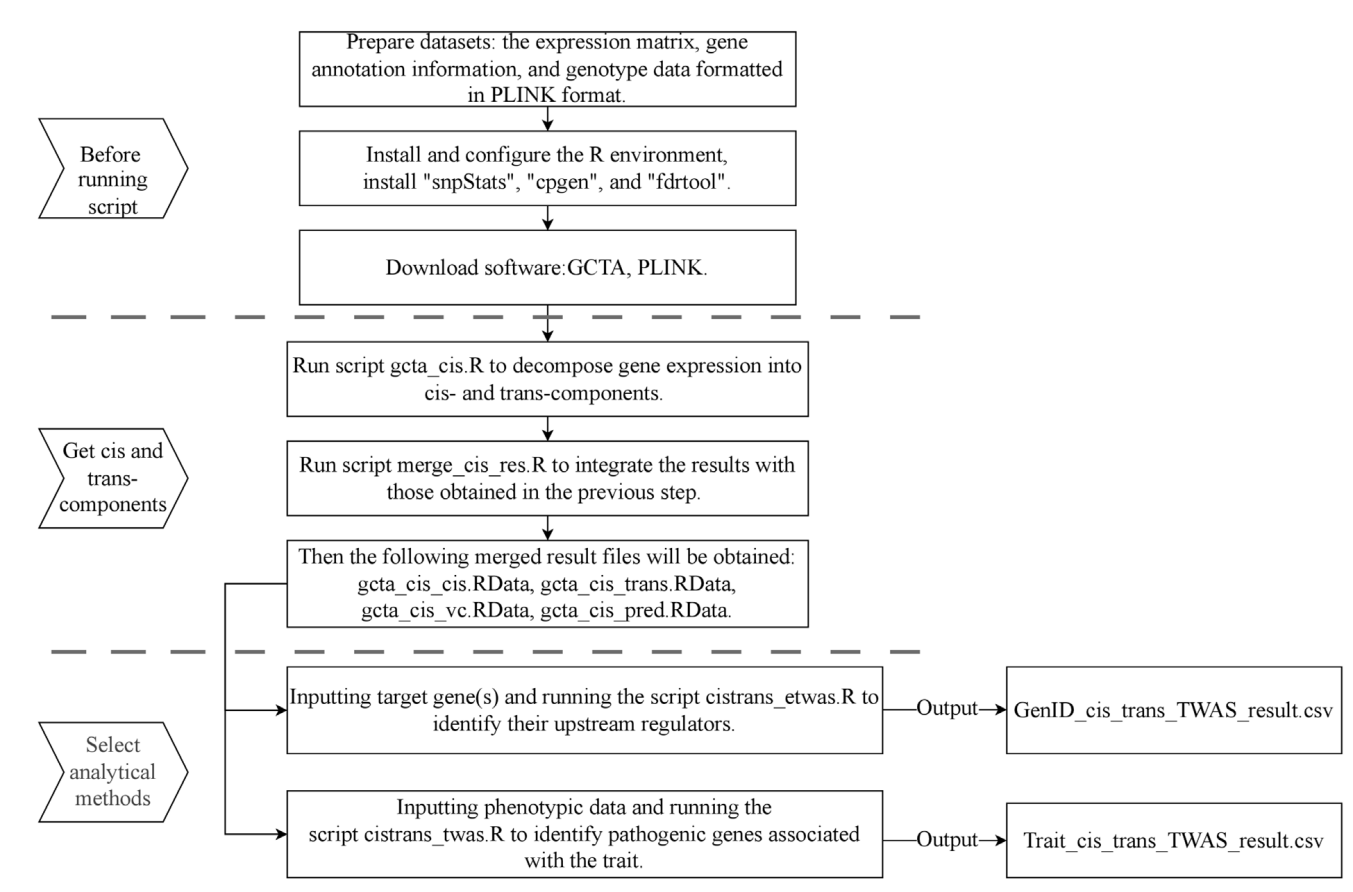


**Flowchart of the CisTrans-ECAS method.** The workflow is divided into three main stages: preparation before running scripts, the process of obtaining *cis*-EC and *trans*-EC, and the selection of analytical methods for downstream analysis. The flowchart details the key scripts (gcta_cis.R, merge_cis_res.R, cistrans_etwas.R, cistrans_twas.R) and their respective inputs and outputs.

## Background

Important agronomic traits in crops, such as yield, quality, and stress resistance, are typically complex traits controlled by multiple genes. Genome-wide association studies (GWAS) are a primary tool for dissecting the genetic basis of these traits. However, in many crops like rice, extensive linkage disequilibrium (LD) and the polygenic nature of traits mean that a single GWAS locus can harbor dozens of genes, making the identification of the true causal gene a formidable challenge. This significantly limits the practical application of GWAS findings.

The advent of transcriptomics has opened new avenues to tackle this issue. Gene expression, as an intermediate layer between genotype and phenotype, is itself under genetic control. Methods like expression quantitative trait loci (eQTL) analysis and TWAS link genetic variants to gene expression levels, providing functional evidence to prioritize candidate genes. Recent TWAS frameworks (e.g., PrediXcan- and FUSION-based approaches) and eQTL-guided colocalization analyses provide functional evidence to link genes with complex traits and have been applied in both human and plant genetics [1–3]. These models predict gene expression from the genotype and test its association with phenotypes, offering a mechanistic layer beyond SNP-trait correlations. Colocalization-based methods further evaluate whether GWAS and eQTL signals share a common causal variant, helping prioritize functional loci. While these approaches have proven valuable, their performance in crops is often hindered by extensive LD, complex population structure, and the difficulty of distinguishing traits driven by local regulation from those influenced by distal regulatory networks.

To address this challenge, we developed the CisTrans-ECAS (*cis-* and *trans-*expression component-based association study) method. Its core concept is that the expression variation of a gene can be partitioned into two components: 1) the *cis-*expression component (*cis*-EC), in which expression variation is explained by local genetic variants (e.g., within 100 kb upstream and downstream); and 2) the *trans-*expression component (*trans*-EC), in which expression variation is driven by distal regulatory factors or other genetic influences not captured by the *cis*-EC.

Unlike traditional TWAS methods that rely on total gene expression or predicted expression levels, CisTrans-ECAS explicitly decomposes expression into *cis-* and *trans-*regulated components. This separation allows the method to distinguish between local regulatory effects and broader regulatory network influences, providing higher specificity for identifying causal genes in regions of extensive LD. Our rationale is that if a gene is a true functional participant in a biological process affecting a trait, its association with the trait should be evident from two independent sources. Its local regulation (*cis-*EC) should be linked to the trait, and its regulation within a broader network (*trans-*EC) should also be associated with the trait.

Therefore, a gene whose *cis-*EC and *trans-*EC are both significantly associated with a target trait is a much stronger causal candidate than a gene with only one type of association. This dual-evidence strategy effectively filters out false positives arising from LD, enabling the precise identification of causal genes, even in regions with weak GWAS signals. Furthermore, by treating the expression of other genes as molecular phenotypes (e-traits), this method can efficiently map upstream and downstream regulatory relationships, providing robust support for establishing reliable gene regulatory networks.

## Software and datasets

**A. Install required software**

This protocol was tested in a Linux server environment. The core analyses rely on PLINK, GCTA, and several R packages (Table 1). While originally developed using R v3.5.1, the protocol is expected to be compatible with recent versions (e.g., R 4.x). We recommend creating a dedicated Conda environment.

1. Create and activate a Conda environment:

# Create and activate a conda environment

conda create -n ecas_env r-base=3.5.1

conda activate ecas_env

2. Install R packages:

Within the activated conda environment, start R and run the following commands to install the required packages.

# Install from CRAN

install.packages("fdrtool")

# Install from conda

conda install bioconda::bioconductor-snpstats

conda install -c conda-forge r-devtools

conda install -c conda-forge r-lme4

# Install from GitHub

devtools::install_github("cheuerde/cpgen", ref = "master", build_vignettes=FALSE)

**Table 1.** Software and resources for data analysis.

Type	Software/dataset/resource	Version	OS	License	Access/source
Software	PLINK	1.9	Linux	GPLv2	https://www.cog-genomics.org/plink/
Software	GCTA	1.93.2beta	Linux	GPLv3	https://yanglab.westlake.edu.cn/software/gcta/
Software	snpStats	1.32.0	R	GPLv2	Bioconductor
Software	cpgen	0.2	R	GPLv3	GitHub
Software	fdrtool	1.2.16	R	GPLv2	CRAN

**B. Companion GitHub repository for protocol implementation**

All scripts, example data, and detailed instructions required for this protocol are available in the following GitHub repository:

• Repository: https://github.com/Minglc/CisTrans-ECAS

• DOI for the code: https://doi.org/10.5281/zenodo.10004834

It is highly recommended to read the README.md file carefully before starting.

## Procedure

**A. Prepare and validate data formats**

Before execution, ensure all input files conform to the formats described in the README.md file. All data frames must be verified to contain valid column headers (header = TRUE) to ensure accurate variable identification and subsequent analysis steps.

str(gffs);str(exp_m);str(genes);str(K);str(pheno)

Key file requirements are summarized below in Table 2.

**Table 2.** Key input parameters and file formats for the CisTrans-ECAS analysis pipeline.

Parameter	Input file format	Content description
--gffs_file	RData	A data frame gffs containing gene annotation and including columns like Gene, chr, start, end, etc.
--exp_file	RData	A gene expression matrix exp_m with genes as rows and individuals as columns. Values should be normalized (e.g., log2-transformed and then quantile-normalized TPMs (transcripts per kilobase million).
--genodir	Pathname	Directory containing genotype data in PLINK binary format (.bed, .bim, .fam), preferably stored per chromosome.
--gfile_prefix	String	The prefix pattern for genotype file names, using %s as a placeholder for the chromosome number, e.g., for chr1_mydata.bed, set this to %s_mydata.
--plinkdir	Pathname	Path to the PLINK executable.
--gctadir	Pathname	Path to the GCTA executable.
--K_file	RData	An RData file containing the kinship matrix K to correct for population structure.
--pheno_file	RData	A data frame pheno containing phenotypic data, with row names as individual IDs and columns for different traits.
--gene_file	RData	A character vector named genes containing a set of target gene IDs for the cistrans_etwas.R analysis.

**B. Decompose gene expression into *cis* and *trans* components**

This step uses GCTA to model gene expression based on *cis*-regulatory SNPs and partitions it into *cis*-EC and *trans*-EC.

1. Calculate expression components per chromosome.

In a Bash environment, execute the gcta_cis.R script for each chromosome.

Rscript gcta_cis.R \

 --gffs_file=./test_data/gffs.RData \

 --exp_file=./test_data/exp_matrix.RData \

 --genodir=./test_data/ \

 --gfile_prefix=%s_529_test \

 --plinkdir=/path/to/plink \

 --gctadir=/path/to/gcta \

 --out_dir=./test_res/gcta_cis \

 --extend=1e5 \

 --ncor=4

*Notes:*

*1. Computational resource benchmark: The decomposition of gene expression using the gcta_cis.R script is the most computationally intensive step of this protocol. Runtime and memory usage depend on the number of individuals, the number of genes per chromosome, the size of the cis-regulatory window (defined by --extend), and the number of CPU cores assigned (--ncor). This script will generate an .RData result file for each chromosome.*

*2. To provide a practical benchmark, we report the resources used for our analysis of a dataset comprising 275 individuals and 30,869 genes. The computation was parallelized by chromosome, with each task assigned to two CPU cores (--ncor=2). The task with the highest demand was for Chromosome 1 (containing 4,146 genes), using a cis-window of 100 kb (--extend=1e5). This single task required approximately 10.5 h to run and had a peak memory usage of 4.2 GB.*

*3. Users should note that these resources are required per parallel task (i.e., per chromosome). The total wall-clock time can be significantly reduced if sufficient CPU cores are available to process all chromosomes simultaneously. We advise users to estimate their needs based on the chromosome with the highest gene count, as it will likely represent the computational bottleneck.*

2. Merge results from all chromosomes.

The per-chromosome outputs from gcta_cis.R must be merged using the merge_cis_res.R script.

Rscript merge_cis_res.R --file_dir=./test_res/gcta_cis

*Note: This step generates four key merged files: gcta_cis_vc.RData (variance components of cis-heritability), gcta_cis_cis.RData (matrix of cis-ECs), gcta_cis_trans.RData (matrix of trans-ECs), and gcta_cis_pred.RData (matrix of cis-predicted expression).*

**C. Associate phenotypes with expression components to prioritize causal genes (cistrans-twas)**

This step performs an association analysis between the calculated *cis*-EC, *trans*-EC, and agronomic traits to identify candidate causal genes.

1. Run cistrans-twas.

Rscript cistrans_twas.R \

 --gffs_file=./test_data/gffs.RData \

 --K_file=./test_data/Kinship.RData \

 --pheno_file=./test_data/pheno.RData \

 --vc_file=./test_res/gcta_cis/gcta_cis_vc.RData \

 --cis_file=./test_res/gcta_cis/gcta_cis_cis.RData \

 --trans_file=./test_res/gcta_cis/gcta_cis_trans.RData \

 --out_dir=./test_res/cistrans_twas \

 --p_threshold=1.62e-6

*Note: The --p_threshold of 1.62 × 10^-6^ corresponds to a Bonferroni-corrected p-value of 0.05 for ~30,869 genes. This value should be adjusted based on the number of genes in your dataset. The reason for this necessary adjustment is that the total number of protein-coding genes in the genome varies significantly among different species, directly impacting the multiple testing burden (N) used in the Bonferroni calculation. It is used to pre-filter genes with significant cis-heritability. This threshold is calculated by dividing the nominal overall significance level (α), typically set at 0.05, by the total number of independent hypothesis tests (N_tests_), which, in this case, corresponds to the number of genes being tested for significant cis-heritability in the dataset. For example, in* H. sapiens, *the number of protein-coding genes typically ranges from ~20,000 to 25,000, with the P_threshold_ spanning from 2.50 × 10^-6^ to 2.00 × 10^-6^.*

2. Interpret the results.

The script generates a .csv result file for each trait. Genes are ranked by their rank_product value, which is the geometric mean of the p-value ranks from the *cis-*EC and *trans-*EC associations. A smaller rank_product indicates a higher likelihood of being a causal gene.

**D. Associate e-traits with expression components to identify regulatory factors (cistrans-etwas)**

This step is used to construct gene regulatory networks by treating the expression of target genes as molecular phenotypes (e-traits).

1. Run cistrans-etwas.

Rscript cistrans_etwas.R \

 --gffs_file=./test_data/gffs.RData \

 --K_file=./test_data/Kinship.RData \

 --exp_file=./test_data/exp_matrix.RData \

 --gene_file=./test_data/genes.RData \

 --vc_file=./test_res/gcta_cis/gcta_cis_vc.RData \

 --cis_file=./test_res/gcta_cis/gcta_cis_cis.RData \

 --trans_file=./test_res/gcta_cis/gcta_cis_trans.RData \

 --out_dir=./test_res/cistrans_etwas \

 --p_threshold=1.62e-6

2. Interpret the results.

For each target gene in the gene_file, a .csv result file is generated. Genes within this file are ranked by rank_product. Top-ranked genes are candidate upstream regulators of the target gene.

The output from the cistrans_etwas.R script is presented below, showing the results for *LOC_Os01g10490*. Each tested gene generates a .csv output file where rows represent associated genes and columns contain gene information and association statistics. To facilitate visualization, the first two rows of the *LOC_Os01g10490* result file have been transposed for display (Table 3). Only the most relevant portion of the table is shown here; the full result file includes additional columns such as chr, start, end, strand, beta_cis, se_cis, zscore_cis, *q-*value_cis, beta_trans, se_trans, zscore_trans, *q-*value_trans, cis_rank, trans_rank, and Qsymbols.

**Table 3.** Example output from cistrans_etwas.R analysis for the target gene *LOC_Os01g10490*.

Gene	*LOC_Os04g02500*	*LOC_Os04g02150*
cis_Vg	0.58	0.54
cis_*p*	5.00E-17	5.00E-17
*p-*value_cis	1.43E-05	2.20E-05
*p-*value_trans	2.32E-28	3.46E-22
rank_product	2	4.47
Note	G-patch domain containing protein expressed	tRNA methyltransferase putative expressed

## Data analysis

This section provides a case study demonstrating how to use the CisTrans-ECAS workflow to identify upstream regulators of the known panicle architecture gene *SDT* (encoding *miR156j*).

1. Define the analysis objective and prepare the input file.

Our biological question is: "Which genes are potential upstream regulators of *SDT* (Gene ID: LOC_Os06g44034) in the rice population?"

To answer this, we create an RData file named genes.RData containing a character vector gene with a single element: "LOC_Os06g44034."

2. Execute the cistrans-etwas analysis.

We use genes.RData as the input for the --gene_file parameter and run the cistrans_etwas.R script as described in section D of the procedure. The script will test for associations between the *cis-*EC and *trans-*EC of all other genes and the expression level of *SDT*.

3. Interpret the output and identify the key candidate regulator.

After the script completes, we identify strong candidate regulators by applying a highly stringent statistical filter to the output file LOC_Os06g44034_cis_trans_TWAS_result.csv. Our criteria require a gene to show significant associations for both its components: a *cis*-EC association p-value < 0.001 and a *trans*-EC association FDR < 0.001. We then use the rank_product statistic to prioritize candidates that meet these thresholds.

Applying these stringent criteria yielded a focused list of only three high-confidence candidate regulators for *SDT*. To illustrate the added value of our method, we compared their final ranking with their ranking based on *cis-*association alone, as summarized in Table 4.

**Table 4.** High-confidence upstream regulators of *SDT* identified by CisTrans-ECAS and comparison with a cis-only ranking. ^a^ Rank_cis is the rank of the gene among all tested genes, sorted by its P-value_cis. ^b^ Rank_trans is the rank of the gene among all tested genes, sorted by its *Q*-value_trans. ^c^ Final_rank is the rank of the significant candidate genes that passed the thresholds, sorted by the rank_product statistic.

Gene ID	Gene symbol	P-value_cis	Rank_cis^a^	*Q*-value_trans	Rank_trans	Final rank^b^
*LOC_Os04g49150*	*OsMADS17*	3.08E-05	26	7.86E-12	1	1
*LOC_Os06g06040*	*OsHOL2*	1.18E-04	44	7.65E-05	78	2
*LOC_Os03g22890*	*OsNug2*	6.08E-04	102	7.77E-04	203	3

This comparison clearly demonstrates the power of the CisTrans-ECAS framework. Notably, all three candidates have modest ranks based on *cis-*association alone (Rank_cis of 26, 44, and 102). A conventional approach relying solely on the strength of *cis-*association would have likely overlooked these genes. Our method, by integrating the highly significant *trans-*association signal, successfully "rescued" these potentially important genes and prioritized them for further investigation.

**Figure 1.**
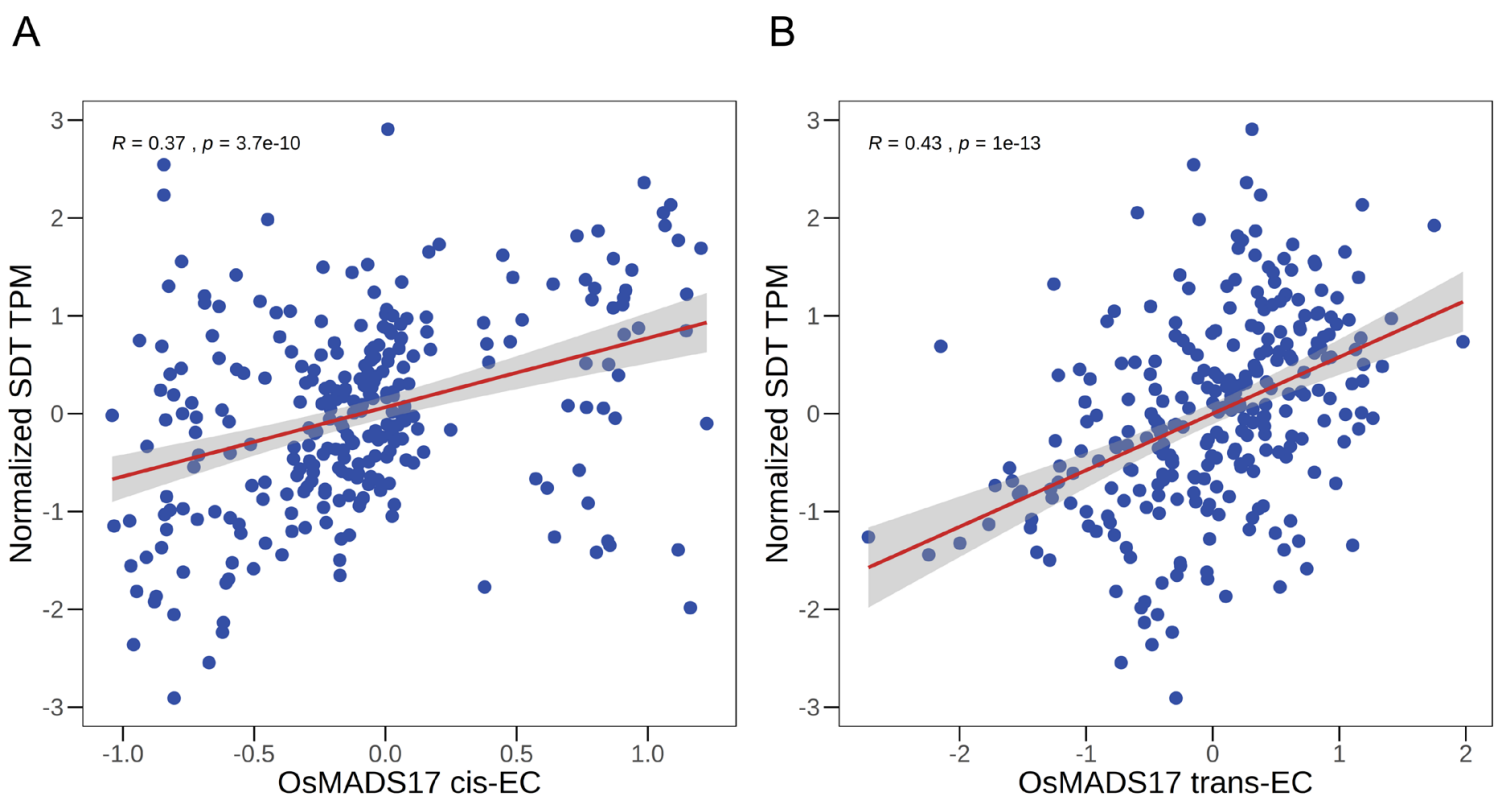
Association between *OsMADS17* expression components and *SDT* expression. (A) Scatterplot showing the positive linear correlation between *cis-*EC of *OsMADS17* and the expression of *SDT*. (B) Scatterplot showing the positive linear correlation between the *trans-*EC of *OsMADS17* and the expression of *SDT*. In both panels, the red line indicates the linear regression fit, and the gray shaded area represents the 95% confidence interval. The Pearson correlation coefficient (R) and corresponding p-value are shown in the top-left side of each plot.

As shown in Table 4, the MADS-box transcription factor OsMADS17 (Gene ID: *LOC_Os04g49150*) emerged as the top-ranked candidate (final rank = 1), making it a prime target for downstream validation. To visualize the dual evidence supporting this top candidate, we plotted the correlation between the *cis*-EC and *trans*-EC values for *OsMADS17* and the raw expression levels of *SDT*, as shown in Figure 1.

**In-depth interpretation of the biological significance**

The results in Figure 1 provide two independent lines of computational evidence supporting the hypothesis that "*OsMADS17* regulates *SDT*":

• Significance of the *cis*-EC association (Figure 1A): *Cis*-genetic variants that regulate the expression of *OsMADS17* itself also have a downstream effect that propagates to *SDT*. This implies a central role for *OsMADS17* in this regulatory cascade.

• Significance of the *trans*-EC association (Figure 1B): Distal genetic factors that regulate *OsMADS17* also affect *SDT*. This suggests that *OsMADS17* and *SDT* likely co-exist within a larger regulatory module.

**Conclusion and methodological advantages**

A traditional eQTL analysis might have missed the *OsMADS17* > *SDT* regulatory link due to insufficient statistical power. By integrating dual evidence from both cis and trans components, the CisTrans-ECAS method greatly enhances the signal-to-noise ratio, enabling the high-confidence capture of this true regulatory event. This computational prediction was subsequently validated by molecular experiments in our published study, demonstrating the power of this protocol in accurately reconstructing gene regulatory networks.

Distinguishing true causal genes from correlated neighbors remains a major challenge in complex trait analysis, especially in crops with extensive LD. The CisTrans-ECAS framework addresses this by requiring independent support from both local regulatory effects (*cis-*EC) and broader network influences (*trans-*EC), effectively filtering out LD-driven false positives. The method accurately identifies previously validated pathways and strongly supports genes such as *SDT* and *OsMADS17* as causal factors, even at small-effect GWAS loci. Genes prioritized by this framework are therefore not merely associated but functionally validated candidates supported by two independent regulatory angles. While association does not imply causation, these findings provide novel insights and valuable resources for elucidating panicle development regulatory networks.

## Validation of protocol

The validity, reliability, and application of this protocol have been systematically confirmed by experiments in our *Nature Communications* article [4].

Key validation points:

1. Successful identification and experimental validation of a causal gene:

• Prediction: Using the cistrans-twas workflow, the method identified *OsMADS17* as a candidate causal gene for spikelets per panicle (SPP) from a locus with weak GWAS signals.

• Validation: CRISPR/Cas9 knockout mutants of *osmads17* exhibited a significant increase in SPP compared to wild type (see Figure 7f–i in the original article), perfectly matching the computational prediction.

2. Accurate prediction and experimental confirmation of a gene regulatory network:

• Prediction: Using the cistrans-etwas workflow, the method predicted that *OsMADS17* acts as an upstream transcriptional activator of *SDT*.

• Validation: Transient expression assays and EMSA confirmed that the OsMADS17 protein directly binds to the *SDT* promoter and activates its transcription (see Figure 7d–e and Supplementary Figure 16 in the original article).

3. Predictive power for phenotypes validated in an independent population:

• Finding: Favorable alleles at the *cis*-regulatory regions of identified candidate genes showed an additive effect. In an independent population, the number of favorable alleles was highly correlated with the corresponding traits (see Figure 6h–i in the original article).

• Significance: This demonstrates that the identified genes and loci have practical value as molecular markers for crop improvement.

## General notes and troubleshooting

**General notes**

1. Data quality is paramount: The performance of this method relies on high-quality genotype, RNA-seq, and phenotype data. We strongly recommend rigorous quality control and normalization of RNA-seq data (e.g., using PEER to correct for hidden confounders).

2. Computational resources: The gcta_cis.R step is computationally intensive. It is advisable to run it on a high-performance computing cluster and parallelize by chromosome. A detailed resource benchmark is provided in section B of the procedure to help users estimate their needs.

3. Parameter selection: The --extend parameter (*cis-*region size) can be adjusted based on the LD decay distance of the species. The --*p*_threshold affects the number of candidate genes and can be adjusted to balance stringency and discovery.

**Troubleshooting**

**Problem 1:** R script fails, reporting a missing package or function.

Possible cause: The R environment is not configured correctly, or dependencies are not fully installed.

Solution: Ensure you have followed the installation instructions in the Software and datasets section precisely. Create the specified conda environment to ensure version compatibility. Verify package installation by loading them one by one in an R session [e.g., library(snpStats)].

**Problem 2:** GCTA or PLINK error during gcta_cis.R execution, with a "command not found" message.

Possible cause: The path specified by --gctadir or --plinkdir is incorrect, or the software lacks executable permissions.

Solution: Use absolute paths to the GCTA and PLINK executables. Ensure they have execute permissions via chmod +x /path/to/gcta.

**Problem 3:** The final number of candidate genes is too large or too small.

Possible cause: The significance thresholds for the association analysis are too lenient or stringent.

Solution: In addition to ranking by rank_product, consider applying a secondary filter based on the FDR-adjusted p-values of cis_p and trans_p. For instance, start with a lenient threshold (e.g., FDR < 0.1) for discovery and then apply a stricter cutoff (e.g., FDR < 0.01) to narrow down top candidates.
